# Eight in One: Hidden Diversity of the Bagrid Catfish *Tachysurus albomarginatus* s.l. (Rendhal, 1928) Widespread in Lowlands of South China

**DOI:** 10.3389/fgene.2021.713793

**Published:** 2021-11-17

**Authors:** Wei-Han Shao, Jian-Li Cheng, E Zhang

**Affiliations:** ^1^ Institute of Hydrobiology, Chinese Academy of Sciences, Wuhan, China; ^2^ College of Advanced Agricultural Sciences, University of Chinese Academy of Sciences, Beijing, China; ^3^ School of Life Sciences, Jinggangshan University, Ji’an, China

**Keywords:** cryptic species, allopatric speciation, integrative taxonomy, species delineation, freshwater fishes

## Abstract

There is increasing evidence that species diversity is underestimated in the current taxonomy of widespread freshwater fishes. The bagrid species *T. albomarginatus* s.l. is mainly distributed in the lowlands of South China, as currently identified. A total of 40 localities (including the type locality), which covers most of its known range, were sampled. Molecular phylogenetic analyses based on concatenated mtDNA and nuclear genes recover nine highly supported lineages clustering into eight geographic populations. The integration of molecular evidence, morphological data, and geographic distribution demonstrates the delineation of *T. albomarginatus* s.l. as eight putative species. Four species, namely, *T. albomarginatus*, *T. lani*, *T. analis*, and *T. zhangfei* sp. nov. and the *T. similis* complex are taxonomically recognized herein. Moreover, *T. zhangfei* sp. nov. comprises two genetically distinct lineages with no morphological and geographical difference. This study also reveals aspects of estimation of divergence time, distribution, and ecological adaption within the *T. albomarginatus* group. The unraveling of the hidden species diversity of this lowland bagrid fish highlights the need for not only the molecular scrutiny of widely distributed species of South China but also the adjustment of current biodiversity conservation strategies to protect the largely overlooked diversity of fishes from low-elevation rapids.

## Introduction

Freshwater ecosystems not only harbor incredible amounts of biodiversity but also provide significant ecosystem services for mankind (Harrison, et al., 2016). However, the threat under which freshwater ecosystems have been put is far greater than that of territorial or marine ecosystems (Darwall, et al., 2018). As an essential composition of this ecosystem, freshwater fishes are currently confronted with severe threats from anthropogenic perturbations (Schmidt et al., 2019). It is therefore urgently needed to take protection actions for freshwater ecosystems in general and for freshwater fishes in particular (Gascón et al., 2008; Rojas et al., 2020). Insufficient basic knowledge regarding species diversity is frequently one of primary impediments for freshwater fish conservation (Darwall et al., 2018). Protection strategies for either freshwater ecosystems or freshwater fishes can become misdirected due to failure to detect the true species diversity, particularly hidden diversity of widespread species. Increasing evidence has shown that widespread species, which are often recognized as a single species owing to very similar or indistinguishable morphology, can comprise multiple species (Das, 2007).

The delineation of hidden diversity within widespread species has the great potential to further our understanding of macroecology, evolutionary processes, and conservation biology (Brickford et al., 2007). The practice of integrative taxonomy can yield many more putative species than traditional taxonomy, which would result in improved biodiversity assessments allowing better modeling of macroecological processes and increased resolution of spatial heterogeneity (Eme et al., 2017). The detection of putative species also has evolutionary significance as they share similar morphology with low rates of phenotypic variation (Tan et al., 2010). Additionally, the average species distribution range decreases drastically with the growing number of species. Species with small range sizes are much more vulnerable to anthropogenic or natural factors than broadly distributed species (Delic et al., 2017). Neglect of the vulnerability of putative species can lead to underestimating threats to biodiversity.

Although the detection of species diversity poses a big challenge for traditional taxonomy, the process of species exploration has been greatly accelerated by the use of current molecular techniques (Kekkonen and Hebert, 2014). Generally, phylogenetic lineages recovered in most cases of molecular analysis can merit their specific status with more or less morphological and/or ecological distinctness (Ozkilinc and Sevinc, 2018). The last decade has witnessed the utilization of molecular technology to unveil the genetic structure differentiations of widely distributed Chinese freshwater fishes, including *Hemicculter leuciclus* (Chen et al., 2017), *Garra orientalis* (Yang et al., 2016), *Squalidus argentatus* (Yang et al., 2013), *Hemibagrus macropterus* (Lei et al., 2009), and *Opsariichthys bidens* (Perdices et al., 2005). Unfortunately, few studies incorporated molecular datasets with morphological evidence to argue for the specific status of recovered genetic lineages. Moreover, it has been revealed that results only from molecular approaches should be utilized with caution for species delineation (Forister et al., 2008). The use of different molecular markers and species delineation methods can lead to conflicting results about the number of delineated species. Seeking congruence between molecular and non-molecular data using integrative taxonomy is currently becoming the principal methodology in species exploration (Welton et al., 2014).

South China lies within the Asian subtropical region with an affluent diversity of freshwater fish species (Du et al., 2021). In stark contrast to the high amount of conservation attention currently given to the highlands of South China, including the upper Yangtze River (= Chang-Jiang in Chinese) and upper Pearl River (=Zhu-Jiang in Chinese) basins, which have high numbers of endemic and threatened fishes (Dao et al., 2020), little or no efforts have been made to protect the non-charismatic fish species of the lowlands of South China such as the mid-lower Chang-Jiang and Zhu-Jiang basins, likely due to a lower level of endemism. However, several widespread freshwater fishes from these lowland areas have been shown to represent multiple distinct genetic lineages by using phylogenetic analysis (Perdices et al., 2005; Yang et al., 2016; Chen et al., 2017), therefore justifying a need to reassess the morphologically based widespread species under a molecular scrutiny. This is the case for the currently identified widespread bagrid species *Tachysurus albomarginatus* from South China.

*Tachysurus albomarginatus* was originally described by Rendahl (1928) on the basis of five 49.0–90.0-mm SL specimens caught from Tang-tu-hsien [now Dangtu County, in the lower Chang-Jiang basin], Anhui Province, South China. The genus *Tachysurus* La Cepède, 1803, with which both *Pseudobagrus* and *Peltobagrus* were synonymized (Ng and Kottelat, 2007), is an endemic East Asian group of small or medium-sized species adapted to the fast-flowing waters of mountain streams or main channels of rivers (Ng and Freyhof, 2007). Although there is ongoing controversy over the specific status of *T. albomarginatus*, the majority of authors since Rendahl have regarded it valid (Chen, 1984; Gao, 1990). In Chinese literature, *T. albomarginatus* is a species widely distributed mainly in the lowlands of South China such as the mid-lower Chang-Jiang, middle Zhu-Jiang, Qiantang-Jiang, and Min-Jiang basins, and all other coastal rivers of Zhejiang and Fujian provinces, with its northern extent at the lower Huang-He and Huai-He basins (note: the suffixes -Jiang and -He in Mandarin Chinese mean river) (Gao, 1990; Cheng and Zhou, 1997). The present identification of this lotic bagrid species is far from satisfactory. Detailed comparisons of available specimens, coupled with a brief review of literature records, showed that *T. albomarginatus*, as conventionally defined, contains multiple species deserving specific recognition (Cheng et al., 2021). A thorough taxonomic revision of this species based on more sufficient sampling is urgently needed.

The objective of this study is to explore the possible hidden species diversity within *Tachysurus albomarginatus* s.l. utilizing integrative approaches. Molecular phylogenetic relationships among intraspecific populations were inferred based on the concatenated mitochondrial and nuclear genes of specimens extensively sampled throughout the majority of its known range. Morphometric measurements and meristic counts were analyzed to test for morphological differentiations among genetic clustering groups. Species delineation within *T. albomarginatus* s.l. was predicated on the congruence between morphological and genetic datasets. The findings of all the above analyses highlight a need for taxonomic reevaluation of currently identified widely distributed species and reconsideration of current protection strategies of freshwater fishes from South China, particularly those found in relatively lower elevations.

## Materials and Methods

### Taxon Sampling, Specimen Collection, and Preservation

**FIGURE 1:**
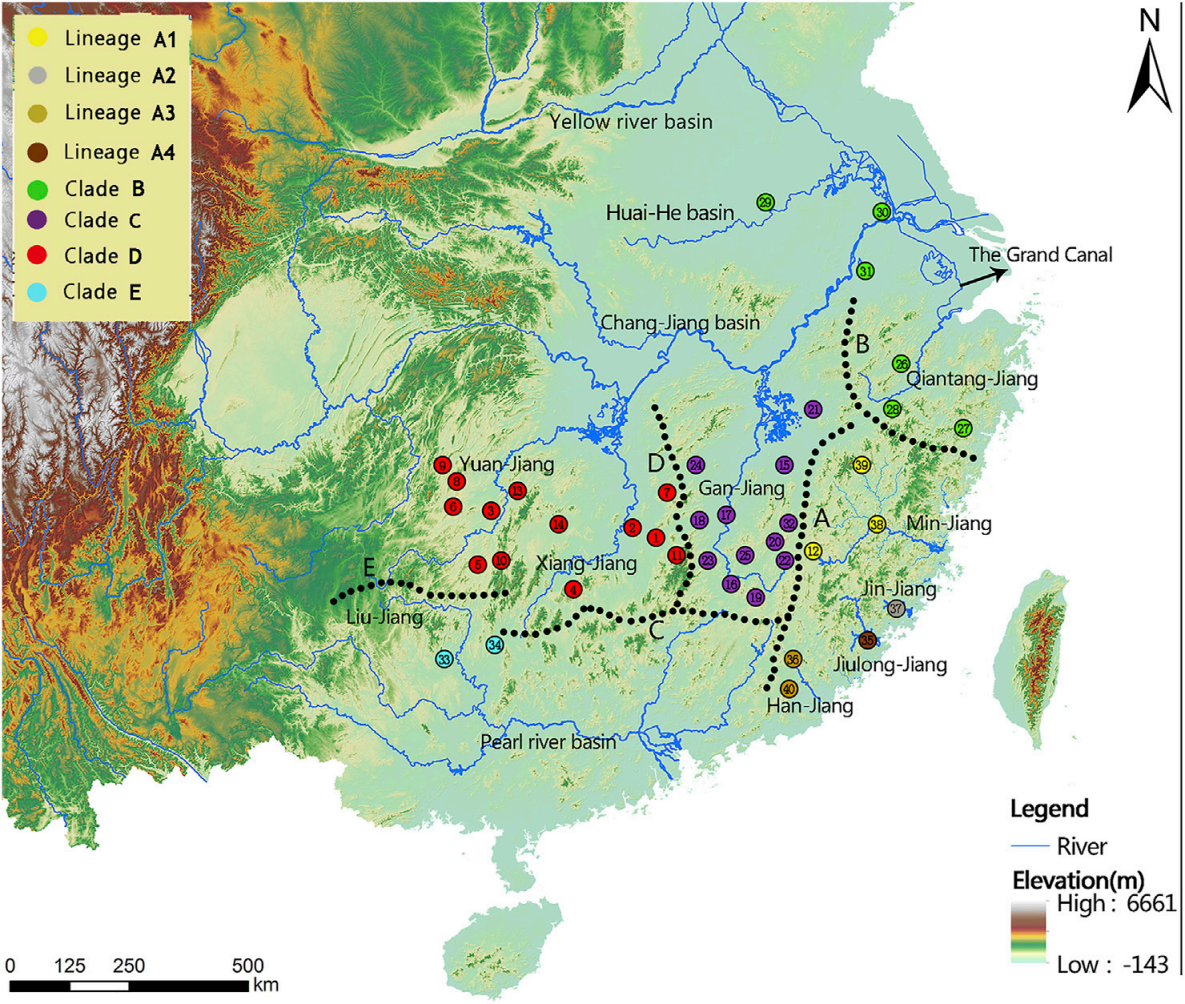
Sampling localities (1-40) and geographical barriers (A–E) within the *T. albomarginatus* species group. Distinct colors denote the same lineage membership as used in Figures 2–6. A, Wuyi Mountains; B, Huangshan-Tianmushan Mountains; C, Nanling Mountains; D, Luoxiao-Mufu Mountains; E, Miaoling Mountains.

Forty sampling locations throughout most of the historically known distribution of *Tachysurus albomarginatus* s.l. were selected, spanning 14 river basins (Figure 1). Sampling localities were numbered from 1 to 40. A total of 418 individuals of the currently recognized *T. albormaginatus* were collected. Among them, 127 individuals were used for molecular analysis, 297 for morphometric analysis, and 207 for meristic counts (see Table 1). Sequences from samples of other 16 congeneric species were also used in the phylogenetic analysis (Table 2), and *Hemibagrus macropterus* was selected as the outgroup.

**TABLE 1:** Detailed sampling drainage with locality and specimen numbers (MCJ: middle Chang-Jiang basin; LCJ: lower Chang-Jiang basin; MZJ: middle Zhu-Jiang basin).

Drainage	No. of sampling localities	No. of specimens		
		Morphometric	Meristic	Molecular
Xiang-Jiang (Dongting Lake, MCJ)	5	19	19	19
Yuan-Jiang (Dongting Lake, MCJ)	8	15	15	11
Gan-Jiang (Poyang Lake, LCJ)	10	33	33	20
Fu-He (Poyang Lake, LCJ)	1	5	5	5
Xin-Jiang (Poyang Lake, LCJ)	1	4	4	4
Liu-Jiang (MZJ)	2	32	25	14
Huai-He	1	31	14	4
Main stem of LCJ	2	16	4	1
Qiantang-Jiang	2	70	18	8
Ou-Jiang	1	4	4	2
Min-Jiang	3	26	26	18
Jin-Jiang	1	8	8	6
Han-Jiang	2	19	19	7
Jiulong-Jiang	1	13	13	8
Total	40	297	207	127

**TABLE 2:** GenBank numbers of sequences used in this study.

Lineages	Haplotype	GenBank number		
		Cyt. b	COI	RAG2
Clade C	Hap10	MT363192	MT379954	MZ636389
	Hap58	MT363193	MT379955	MZ636390
	Hap20	MT363194	MT379956	MZ636391
	Hap48	MT363195	MT379957	MZ636392
	Hap24	MT363196	MT379958	MZ636393
	Hap27	MT363197	MT379959	MZ636394
	Hap25	MT363198	MT379960	MZ636395
	Hap26	MT363199	MT379961	MZ636396
	Hap37	MT363200	MT379962	MZ636397
	Hap57	MT363201	MT379963	MZ636398
	Hap12	MT363202	MT379964	MZ636399
	Hap13	MT363203	MT379965	MZ636400
Lineage D1	Hap46	MT363204	MT379966	MZ636401
	Hap47	MT363205	MT379967	MZ636402
	Hap15	MT363206	MT379968	MZ636403
	Hap14	MT363207	MT379969	MZ636404
	Hap16	MT363208	MT379970	MZ636405
	Hap9	MT363209	MT379971	MZ636406
	Hap60	MT363210	MT379972	MZ636407
	Hap54	MT363211	MT379973	MZ636408
	Hap45	MT363212	MT379974	MZ636409
Lineage D2	Hap6	MT363213	MT379975	MZ636410
	Hap44	MT363214	MT379976	MZ636411
	Hap8	MT363215	MT379977	MZ636433
	Hap19	MT363216	MT379978	MZ636412
	Hap18	MT363217	MT379979	MZ636413
	Hap7	MT363218	MT379980	MZ636414
Clade E	Hap29	MT363219	MT379981	MZ636415
	Hap28	MT363220	MT379982	MZ636416
	Hap56	MT363221	MT379983	MZ636417
Lineage A1	Hap36	MT363222	MT379984	MZ636418
	Hap34	MT363223	MT379985	MZ636419
	Hap49	MT363224	MT379986	MZ636420
	Hap50	MT363225	MT379987	MZ636421
	Hap53	MT363226	MT379988	MZ636422
	Hap51	MT363227	MT379989	MZ636423
	Hap52	MT363228	MT379990	MZ636424
	Hap33	MT363229	MT379991	MZ636425
	Hap35	MT363230	MT379992	MZ636426
Lineage A2	Hap40	MT363231	MT379993	MZ636427
Lineage A3	Hap30	MT363232	MT379994	MZ636428
	Hap31	MT363233	MT379996	MZ636429
	Hap32	MT363234	MT379995	MZ636430
Lineage A4	Hap59	MT363235	MT379997	MZ636431
Clade B	Hap41	MT379998	MT363236	MZ636380
	Hap43	MT379999	MT363237	MZ636381
	Hap23	MT380000	MT363238	MZ636382
	Hap22	MT380001	MT363239	MZ636383
	Hap21	MT380002	MT363240	MZ636384
	Hap38	MT380003	MT363241	MZ636385
	Hap42	MT380004	MT363242	MZ636386
	Hap11	MT380005	MT363243	MZ636432
	Hap17	MT380006	MT363244	MZ636387
	Hap55	MT380007	MT363245	MZ636388
Congeneric species		MZ636449	MZ636463	MZ636476
*Tachysurus tenuis*		MZ636450	MZ636464	MZ636477
*Tachysurus ussuriensis*		MZ636451	MZ636465	MZ636478
*Tachysurus gracilis*		MZ636452	MZ636466	MZ636479
*Tachysurus barchyrhabdion*		MZ636453	MZ636467	MZ636480
*Tachysurus pratti*		MZ636454	MZ636468	MZ636481
*Tachysurus kypus*		MZ636455	MZ636469	MZ636482
*Tachysurus virgatus*		MZ636456	MZ636470	MZ636483
*Tachysurus ondon*		MZ636457	MZ636471	MZ636484
*Tachysurus intermedius*		MZ636458	MZ636472	MZ636485
*Tachysurus eupogon*		MZ636459	MZ636473	MZ636486
*Tachysurus argentivittatus*		MZ636460	MZ636474	MZ636487
*Tachysurus trillineatus*		MZ636461	MZ636475	MZ636488
*Tachysurus sinensis*		MZ636439	MZ636434	MZ636444
*Tachysurus nutidus*		MZ636440	MZ636435	MZ636445
*Tachysurus truncatus*		MZ636441	MZ636436	MZ636446
*Tachysurus vachelli*		MZ636442	MZ636437	MZ636447
Outgroup				
*Hemibagrus macropterus*		MZ636443	MZ636438	MZ636448

### DNA Extraction, Amplification, and Sequencing Analyses

Genomic DNA was extracted from alcohol-preserved fin clips using the DNeasy Tissue Kit (Qiagen, Beijing, China). Two mitochondrial genes [cytochrome b (Cyt. b) and cytochrome *c* oxidase subunit I (COI)] and one nuclear gene [recombination activating 2 (RAG2)] were amplified using polymerase chain reaction (PCR). The information for the primers used in this study is shown in Table 3. The following thermal cycling profiles were adopted: 95°C predenaturing (5 min), 95°C dematuring (30 s), 58°C for RAG2 or 52°C for Cyt. b and COI annealing (45 s), 72°C extension (90 s), for 35 cycles, and 72°C final extension (8 min). Amplified products were subsequently purified and utilized for direct cycle sequencing by Tianyi Huiyaun Sequencing Company. Sequence data were archived in the public domain database GenBank (Table 2). Multiple alignments were prepared utilizing MEGA 7.0 for all sequences, based on the amino acid sequences with the program MUSCLE (Edgar, 2004) with the default setting.

**TABLE 3:** Primers used for both PCR amplification and sequencing in the present study.

Primer	Primer sequences (5′-3′)	Locus	References
L14724	GAC​TTG​AAA​AAC​CAC​CGT​TG	Cyt. b	Xiao et al. (2001)
H15915	CTCCGATCT CCGGATTACAAGAC	Cyt. b	Xiao et al. (2001)
COIF	CTACAATCACCGCC TAAR	COI	Liang et al. (2016)
COIR	TAG​AAG​AAA​GTG​ACA​GAG​CG	COI	Liang et al. (2016)
MHF1	TGyTATCTCCCACCTCTGCGyTACC	RAG2	Dawar et al. (2019)
MHR1	TCATCCTCCTCATCkTCCwTTFTA	RAG2	Dawar et al. (2019)

### Phylogenetic Analyses

COI, Cyt. *b*, and RAG2 genes of each analyzed specimen were concatenated to form a dataset for phylogenetic tree reconstructions with Bayesian inference (BI) and Maximum likelihood (ML). The general time-reversible model with invariant sites and a gamma distribution variation across sites (GTR + G) was selected as the best-fitting model for COI, Cyt. *b*, and RAG2 in Jmodeltest2 (Darriba et al., 2012) based on Akaike informative criterion (Akaike, 1974). MrBayes 3.1.2 (Ronquist and Huelsenbeck, 2003) was applied to calculate BI. Two independent parallel Markov chain Monte Carlo runs with four chains for 50,000,000 generations were performed, sampling every 1,000 generations and discarding the first 25% samples as burn-in. Sufficient convergence of the Markov chain Monte Carlo (MCMC) results was verified based on summary statistics in MrBayes v3.2.6. A maximum likelihood tree of the concatenated dataset was generated with RAxML GUI v.1.5b, applying GTR + G to both COI and Cyt. *b* (Silvestro & Michalak, 2012). Bootstrap values (BS) of nodes were estimated by 1,000 bootstrap replicates. All trees were rooted by *Hemibagrus macropterus* and visualized in FigTree v1.4.3 (http://tree.bio.ed.ac.uk/software/figtree/).

Haplotype networks were constructed for the concatenated mtDNA sequence datasets to better visualize relationships in multifurcations and reticulations (Posada and Crandall, 2001). A maximum parsimony method, tcs v.1.23 (Clement et al., 2000), was used to draw an unrooted network to evaluate the haplotype relationships for the mtDNA sequences, with 95% parsimoniously plausible branch connections. The genetic distance based on the K2P (Kimura two-parameter) model (Kimura, 1980) was calculated with MEGA 7.0. DNASP v5 was used to filter the haplotype (Librado and Rozas, 2009).

### Species Delineation

Two independent methods relying on different operational criteria were applied to infer molecular species delineation for *T. albomarginatus* s.l.: the automatic barcode gap detection (ABGD) and the Poisson tree process (PTP) model. The automatic barcode gap discovery (ABGD) is a barcode species delineation method aiming to establish a minimum gap that probably corresponds to the threshold between interspecific and intraspecific processes (Puillandre et al., 2012). The major advantage of the ABGD method, when compared to the other barcode species delineation ones, is that the inference of the limit between interspecific and intraspecific processes is recursively applied to previously obtained groups to get finer partitions until there is no further partitioning, thus allowing a more refined search. Basically, the ABGD analysis indicates the number of groups (species) estimated relative to a large spectrum of *p* values (prior intraspecific values) in which a 0.1 value assumes a maximum intraspecific variability, suggesting that all sequences belong to only one species, whereas a 0.001 value assumes a very small intraspecific variability suggesting that each distinct haplotype represents a different species. After performing the ABGD analysis, additional molecular, morphological, or ecological characters are needed to infer the correct number of species. The ABGD analysis was run on the ABGD server website (https://bioinfo.mnhn.fr/abi/public/abgd/abgdweb.html).

The PTP is a tree-based species delineation method under the assumption that the intraspecific substitution number is significantly less than the interspecific substitution number (Kapli et al., 2017). The Bayesian (PTP) and maximum likelihood (mPTP) implementations from concatenated data were used in our study. The branch lengths of the phylogenetic input tree represent the number of substitutions. Unrooted phylogenetic trees, without outgroup, created by MrBayes and RAxML were uploaded to the online server of PTP and mPTP (http://species.h-its.org/ptp/).

### Divergence Time Estimation

We estimated the divergence time of *T. albomarginatus* s.l. using the birth/death speciation process model in Beast v1.10.4 with concatenated data of Cyt. *b* and COI sequences (Drummond and Rambaut, 2007). Best-fit models of Cyt. *b* and COI were applied in estimation of divergence time. We adopted a substitution rate of 0.18–0.30% per million years for cyt. *b*, which is proposed by Peng et al. (2002) for the East Asian bagrid catfishes. The uncorrelated log-normal relaxed clock was applied to Cyt. *b* and COI. The mean and standard deviation of the Cyt. *b* substitution rate, i.e., its ucldMean and ucldStdev, were fixed to 0.0024 and 0.136, setting a 95% highest posterior density interval (HPD95) to 0.18–0.30% per million years for substitution rate. The mean rate and standard deviation of COI were estimated in the MCMC run. A single MCMC run was performed with 200,000,000 generations, sampling every 1,000 generations. Sufficient convergence and burn-in of runs were checked in Tracer v1.7 (http://tree.bio.ed.ac.uk/software/tracer) to ascertain that all effective sampling size (ESS) values are above 200. The first 10% of samples were discarded as burn-in in TreeAnnotator v1.10.4 in the Beast package (Drummond and Rambaut, 2007). A consensus tree was generated with maximum clade credibility. The consensus tree was visualized in FigTree v1.4.3 (http://tree.bio.ed.ac.uk/software/figtree/).

### Morphological Analysis

Twenty-six morphometric measurements utilized in this study are presented in Table 6. These measurements were made on the left side of each individual wherever possible. Following the methods of Cheng et al. (2008), data were taken point to point with digital calipers linked to a data recording computer and recorded to the nearest 0.1 mm. Morphometric measurements were subject to principal component analysis (PCA) in order to check external morphological differentiation and explore the relative contributions of specific variables to morphological differences in the target species.

PCA was run with SPSS 16 (SPSS, Chicago, IL, USA). Prior to the analysis, the method of Reist (1985) was used to normalize all morphometric measurements except standard length to eliminate the influence of allometry of body parts and sample size on the morphometric data. The formula of the corrected measurements was given as follows: Mtrans = log M-b (log BL-log BLm) where Mtrans is the size-transformed measurement for each individual; M is the original unadjusted measurement; b is the allometric coefficient that was calculated as the slope of log M against log BL; BL is the standard length of each individual; and BLm is the overall mean standard length of one population while log is the base 10 logarithm. All measurements except standard length were transformed separately utilizing the regression slope and common overall mean body length. The PCA loadings are presented in Table 6.

The counts of vertebrae were taken from photographs of Micro-CT (X-ray-based micro-computed tomography) scanning to explore internal morphology. Micro-CT scanning was made in a Siemens SOMATOM Definition X-ray machine (120 kV, 400 mA, and 0.4 mm slice thickness and voxel dimensions 0.16 mm × 0.16 mm×0.16 mm). Micro-CT data were imported into the Mimics medical imaging software (Materialise N.V., Leuven, Belgium), and a 3D digital reconstruction of each block was created. The individual reconstructions were tidied, smoothed, and stitched together utilizing Adobe Photoshop (Adobe Systems Inc., Mountain View, CA, USA).

## Results

### Sequence Characteristics

The sequence information for the collection used in the molecular phylogenetic analysis is shown in Table 4. A total of 3,497 bp nucleotides were sequenced for one nuclear and two mitochondrial genes: RAG 2 (944 bp), COI (1,438 bp), and Cyt. b (1,115 bp). The nucleotide sequences of these three protein-coding genes code for 314, 479, and 371 amino acids, respectively. For the whole dataset, 401 characters are variable and 162 characters are parsimony informative. The variable and parsimony sites of the RAG2 gene are both fewer than those of CO1 and Cyt. b, thus suggesting the former as being less informative when compared to the latter two mitochondrial genes.

**TABLE 4:** Sequence characteristics of different markers.

Marker	Sites included	Variable sites			Parsimony informative sites (in %)			G	C	T	A
		1st	2nd	3rd	1st	2nd	3rd				
COI	1,438	50	14	134	29 (8.19)	6 (3.39)	52 (14.69)	23.65	19.87	27.44	29.04
Cyt. b	1,115	44	13	113	21 (13.91)	4 (2.65)	35 (23.18)	24.78	25.62	19.86	29.75
Rag 2	944	9	5	19	4 (8.33)	3 (6.25)	8 (16.67)	22.18	25.89	27.77	24.16

### Phylogenetic Analysis and Genetic Distances

**FIGURE 2:**
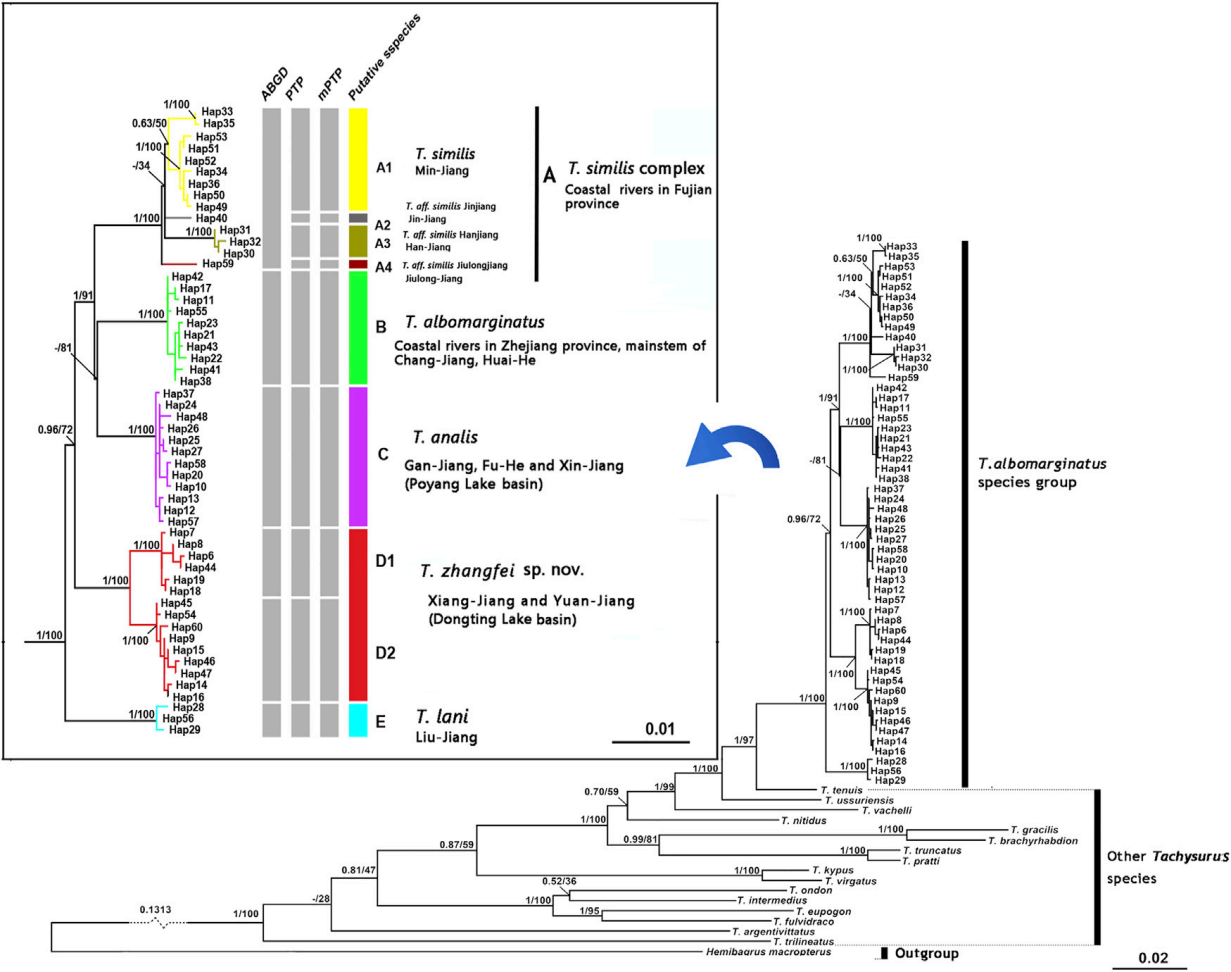
Bayesian tree based on combined genes for the *T. albomarginatus* species group. Values on the left of the nodes indicate posterior probabilities and bootstrap supports of Bayesian inference/maximum likelihood for major lineages. The results of species delimitation are shown on the right.

Phylogenetic trees for *T. albomarginatus* s.l. are inferred from the combined mtDNA (COI and Cyt. b) and nuclear (RAG2) genes using two different inference approaches (ML and BI). Analyses based on these two approaches results in the same general topology (Figure 2). All samples here recognized as *T. albomarginatus* s.l. constitute a monophyletic assemblage strongly supported by a 100% bootstrap value (hereafter bv) and posterior probability (hereafter pp) and cluster into five clades (A, B, C, D, and E) well supported by 100% bv and 100% pp. While Clades B, C, and E include a single lineage (hereafter also called Lineage B, C, or E for convenience of comparison), Clades A and D comprise four and two lineages, respectively. Clade E is the earliest diverging lineage including samples from the Liu-Jiang of the Zhu-Jiang basin. It is supported by 96% bv and 72% pp to be sister to the group formed by the remaining four clades where Clade D is the basal lineage and the weakly supported (less than 50% bv and 81% pp). Paired clades (B and C) receive a strong support of 100% bv and 91% pp to be sister to Clade A. Clade D is constituted by samples from the Yuan-Jiang and Xiang-Jiang, two affluents of Lake Dongting linked to the middle Chang-Jiang, and comprises two sister lineages (D1 and D2), sympatrically occurring or having a entirely overlapping distribution. Clade C is made up of samples from the Xin-Jiang, Fu-He, and Gan-Jiang, three affluents of Lake Poyang connected with the mainstem of the lower Chang-Jiang. Clade B is composed of samples from the lower Huai-He basin, the main stream of the lower Chang-Jiang, and coastal river basins of Zhejiang Province such as the Qiantang-Jiang and Ou-Jiang. There are four lineages (A1-A4) clustered within Clade A where Lineage A4 is sister to the remaining three ones constituting a polytomy. These lineages are each weakly supported but exhibited a significant regional clustering as they correspond to four coastal river basins of Fujian Province. Lineages A1-A4 are each found only in the Min-Jiang, Jin-Jiang, Han-Jiang, and Jiulong-Jiang basins, respectively.

**FIGURE 3:**
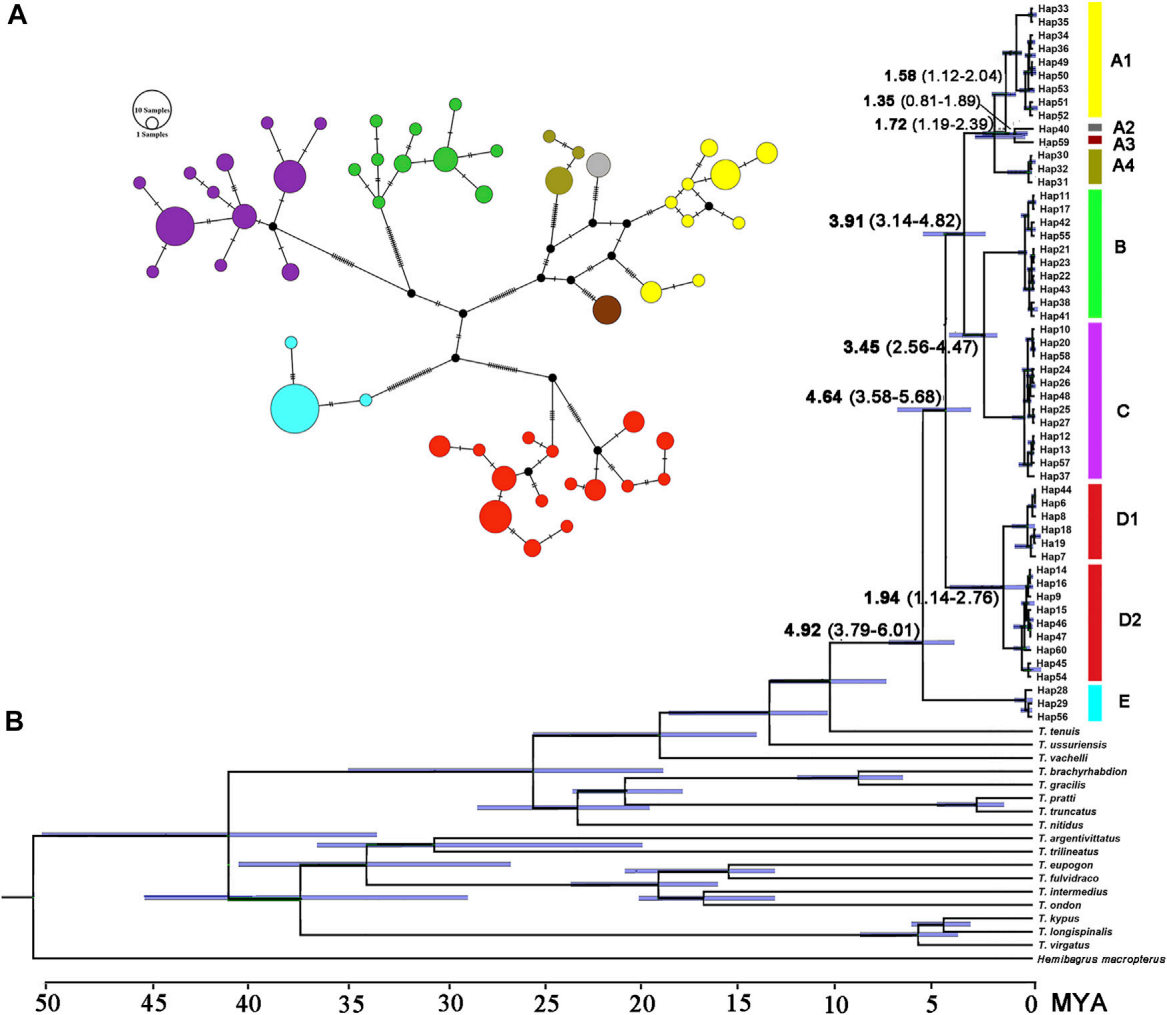
**(A)** Median-joining network (each circle represents a haplotype proportional to its frequency colored by the genetic split in the phylogenetic tree) and **(B)** the chronogram (BEAST) of the main divergence events in *T. albomarginatus* s.l. inferred for the concatenated Cyt. b and COI data.

The paired Clades A and B and four lineages within Clade A receive a low nodal support and have short branch length in the molecular phylogenetic tree of the *T. albormarginatus* s.l. species group (Figure 2); however, the median-joining network analysis corroborates the split of nine lineages (A1, A2, A3, A4, B, C, D1, D2, and E) recovered here and no haplotype is found to be shared among these lineages (Figure 3). Besides, four lineages (A1-A4) within Clade A form network-like patterns instead of dichotomic cladogenesis in the haplotype network analysis.

The estimated K2P genetic distances of the Cyt. b gene between *T. albomarginatus* s.l. and congeneric species range from 5.6 to 12.5% and those between paired lineages within *T. albomarginatus* s.l. range from 1.1 to 2.4% (Table 5). The mean genetic distance among these lineages is 1.7%, and the range of intraspecific genetic distances within each lineage is 0.1–0.2%. Four allopatric lineages (Lineages A1 to A4) from the coastal rivers of Fujian Province exhibit K2P genetic distances from 1.1 to 1.3%, while sympatric paired Lineages D1 and D2 from the Xiang-Jiang and Yuan-Jiang have a 2.2% genetic distance. The K2P distances of COI among/within nine lineages are at the range of 1.2–2.5%/0.1–0.2%. There is relatively less genetic divergence for nuclear genes than for mitochondrial genes, and K2P distances of RAG2 among nine lineages are ranged at 0.1–0.3%.

**TABLE 5:** K2P distances for MOTUs within the *T. albomarginatus* s.l. based on the Cyt. b gene.

Genetic lineages	1	2	3	4	5	6	7	8
1. Clade C								
2. Lineage D1	0.023							
3. Lineage D2	0.022	0.022						
4. Clade E	0.023	0.022	0.023					
5. Lineage A1	0.021	0.023	0.024	0.022				
6. Lineage A2	0.020	0.021	0.021	0.021	0.011			
7. Lineage A3	0.019	0.021	0.022	0.022	0.012	0.013		
8. Lineage A4	0.022	0.020	0.021	0.020	0.013	0.011	0.012	
9. Clade B	0.024	0.022	0.023	0.022	0.021	0.023	0.022	0.020

### Divergence Time Estimate

The estimated time, with a 95% highest posterior density (HPD), is shown in Figure 3B. The most recent common ancestor of the *T. albomarginatus* group was estimated to be around 4.92 Ma (95% HPD: 3.79–6.01) (Figure 3B). Following the divergence of Clade E, Clade D branched off from the remaining clades 4.64 Ma (95% HPD: 3.56–5.68). The time that Clade A separated from the paired Clades B and C was about 3.91 Ma (95% HPD: 3.14–4.82). The diverging time between Clades B and C was inferred to be about 3.45 Ma (95% HPD: 2.56–4.47). Within Clade A, Lineage A4 deeply split 1.72 Ma (95% HPD: 1.19–2.39); the subsequent divergence of Lineage A1 occurred about 1.58 Ma (95% HPD: 1.12–2.04), Lineage A2 and A3 diverged 1.35 Ma (95% HPD: 0.81–1.69).

### Molecular Species Delineation

The ABGD analysis supports six molecular operational taxonomic units (MOTUs) at a prior maximal distance of 0.0100, and these six MOTUs correspond to four clades (A, B, C, and E) and two lineages (D1 and D2), recovered in the molecular phylogenetic analysis based on the combined mtDNA and nuclear genes (Figure 2). Concurrently, the PTP analysis identifies nine MOTUs with the posterior probabilities (higher than >0.70) of 0.90, 0.88, 0.99, 0.99, 0.91, 0.96, 0.89, 0.90, and 0.92; these MOTUs correspond to three clades (B, C, E) and six lineages (A1, A2, A3, A4, D1, and D2). The same result is repeated in the mPTP analysis where the recognition of these MOTUs is supported with the posterior probabilities of 0.91, 0.92, 0.99, 0.95, 0.98, 0.93, 0.92, 0.90, and 0.88, respectively. The difference in species delineation between these two methods is that four lineages (A1-A4), clustered within Clade A from four coastal river basins of Fujian Province, are delimited by the ABGD method as only one MOTU and by the PTP method as four MOTUs.

### Morphological Distinctions

**FIGURE 4:**
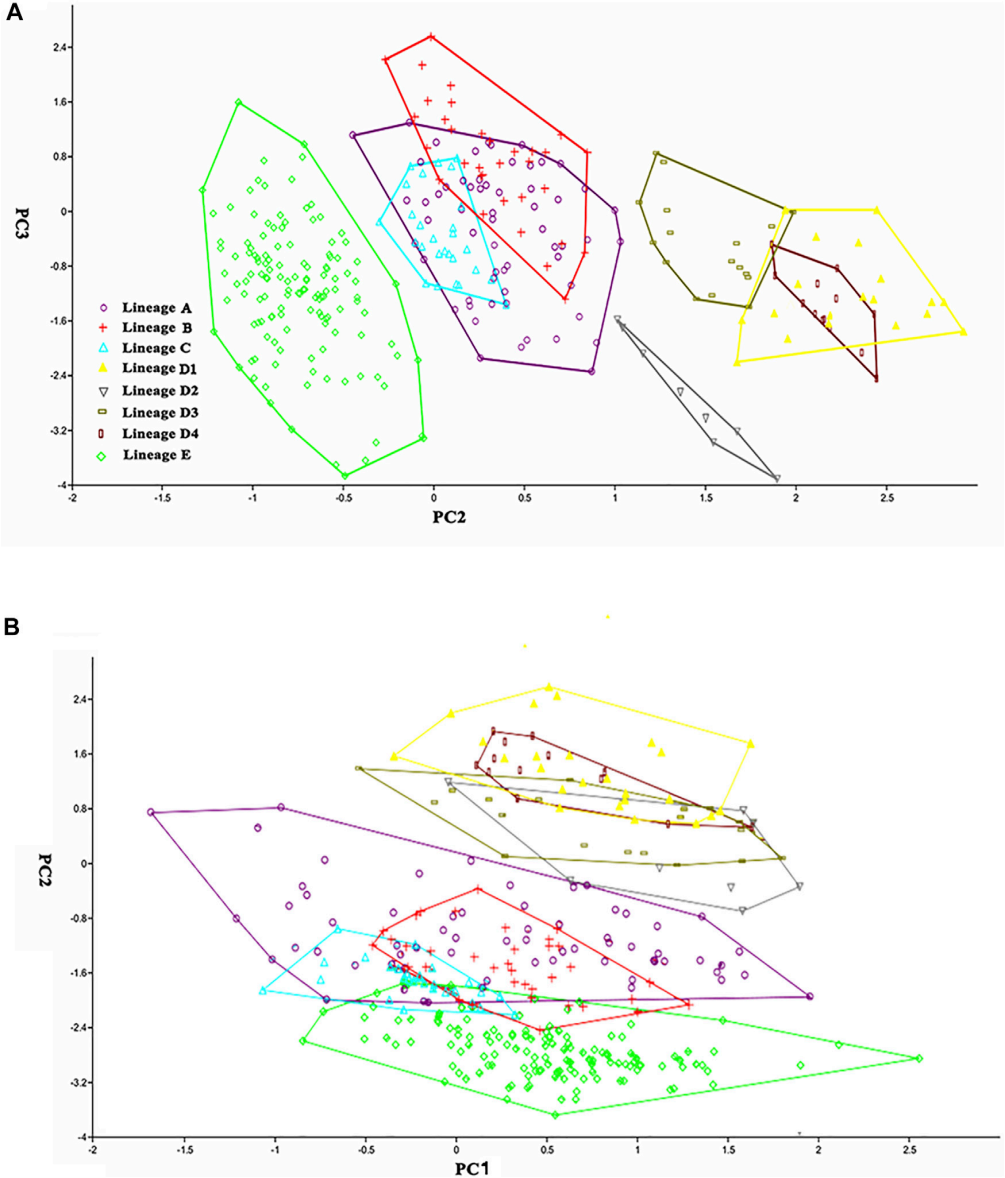
Scatter plot of **(A)** PC2 against PC3 and **(B)** PC1 against PC2 extracted from morphometric data for eight geographic populations.

The nine distinct lineages detected in the molecular phylogenetic analysis correspond to eight geographical populations (Figure 2). Paired lineages D1 and D2 are coexisting and form a single geographical population. Each of the remaining seven lineages (A1, A2, A3, A4, B, C, E) represents its own geographical population. The PCA performed for 26 morphometric measurements (Table 6) show, to some extent, variations among these eight geographic populations. The scatter plot of PC2 versus PC3 calculated for eight populations indicates that they cluster into four groups (Figure 4A). Clade B and Lineage A2 each form a distinct group completely separating from all other groups. Clades C, D, and E are grouped together, largely overlapping with each other, and so does Lineages A1, A3, and A4. Lineages A1 and A4 cluster together or are unseparated but slightly overlap with Lineage A3. The sympatric paired Lineages D1 and D2 are not distinguishable in either morphometric data or meristic counts (Supplementary Figure S1). The scatterplot of PC1 vs. PC2 shows the similar pattern with that of PC2 vs. PC3 except that Lineages A1-A4 heavily overlap with each other (Figure 4B).

**TABLE 6:** Variable loadings in the first to third axes of the principal component analysis among eight allopatric populations within *T. albomarginatus* s.l.

	PC1	PC2	PC3
Standard length	−0.205	0.133	−0.131
Body depth at anus	−**0.216**	−0.092	0.02
Pre-dorsal length	−0.19	0.120	0.008
Pre-anal length	−0.196	0.110	−0.041
Pre-pelvic length	−0.197	0.108	−0.033
Pre-pectoral length	−0.184	0.118	−0.077
Dorsal-fin spine length	−0.205	0.083	0.189
Dorsal-fin base length	−0.194	0.140	0.019
Pectoral-fin spine length	−0.192	0.149	0.162
Pelvic-fin length	−0.187	0.119	0.032
Anal-fin base length	−**0.219**	0.190	−0.207
Adipose-fin height	−0.157	−0.120	0.021
Adipose to caudal distance	−0.211	0.260	−**0.404**
Caudal-peduncle length	−0.211	0.153	−**0.381**
Caudal-peduncle depth	−0.212	−0.103	0.007
Head length	−0.181	−0.090	−0.114
Head depth	−0.194	−**0.711**	−0.311
Head width	−0.178	−0.048	0.039
Snout length	−0.207	−**0.355**	−0.002
Interorbital width	−0.190	0.006	0.05
Eye diameter	−0.159	0.101	0.172
Mouth width	−0.194	0.003	−0.1
Nasal-barbel length	−0.198	−0.0712	0.151
Maxillary-barbel length	−0.197	−0.181	0.137
Inner mandibular-barbel length	−0.210	0.070	**0.439**
Outer mandibular-barbel length	−0.199	−0.111	**0.414**
Percentage of total variances	82.83%	5.41%	2.64%

There are two morphometric measurements (anal-fin base length and body depth at anus) with main loadings on the first axis (PC1), three (adipose to caudal distance, head depth, and snout length) on the second axis (PC2), and two (inner and outer mandibular-barbel lengths) on the third axis (PC3) (Table 6). Stable differences in the head depth, outer mandibular-barbel length, and body depth at the anus are found among these lineages (Figure 6). Counts of vertebrae and anal-fin rays are found to distinguish among them (Figure 4). These eight geographical populations can be clearly distinguishable through the combination of morphometric measurements and meristic counts.

**FIGURE 5:**
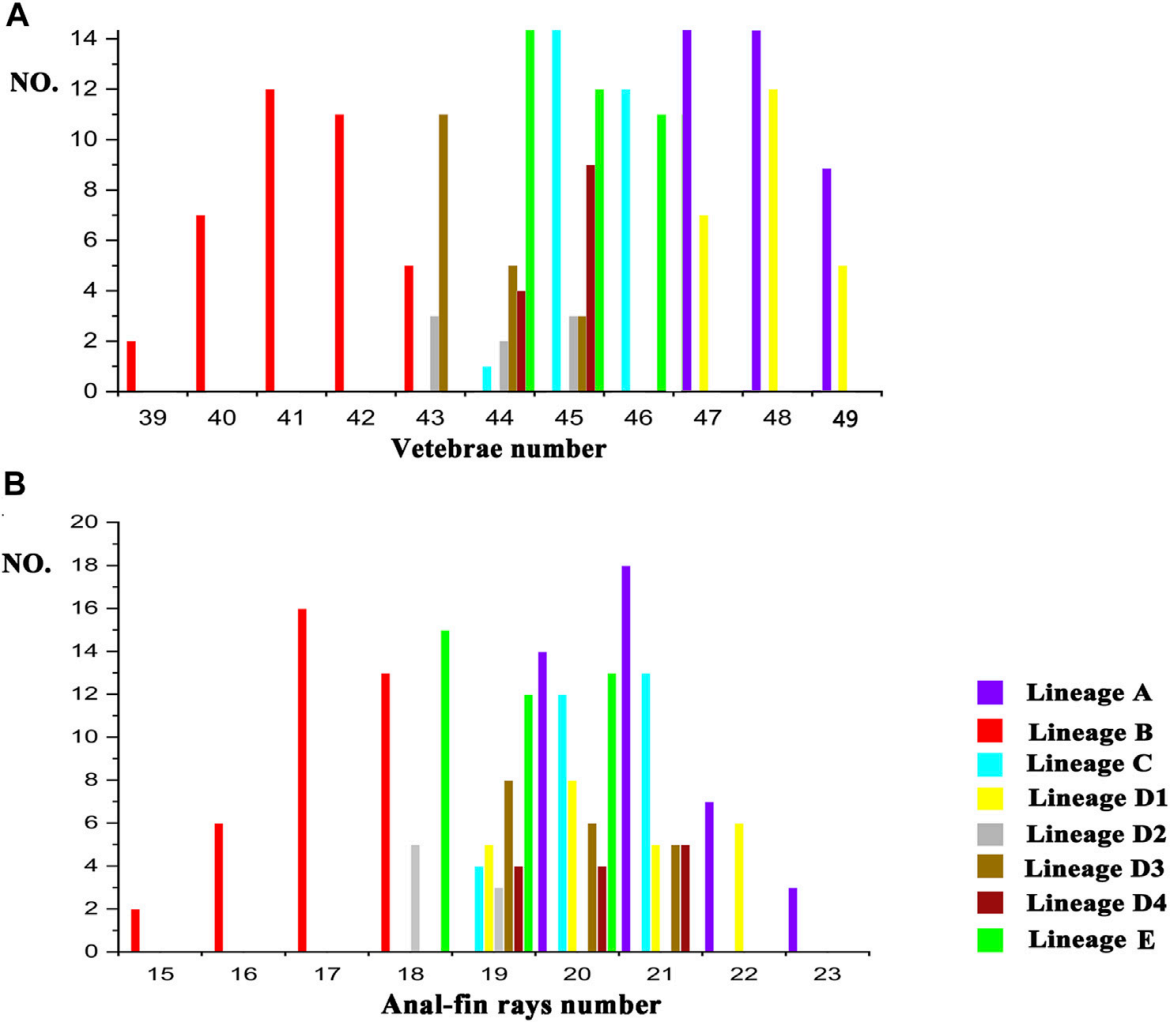
Meristic counts difference among eight geographic populations in vertebrae number **(A)** and anal-fin ray number **(B)**.

**FIGURE 6:**
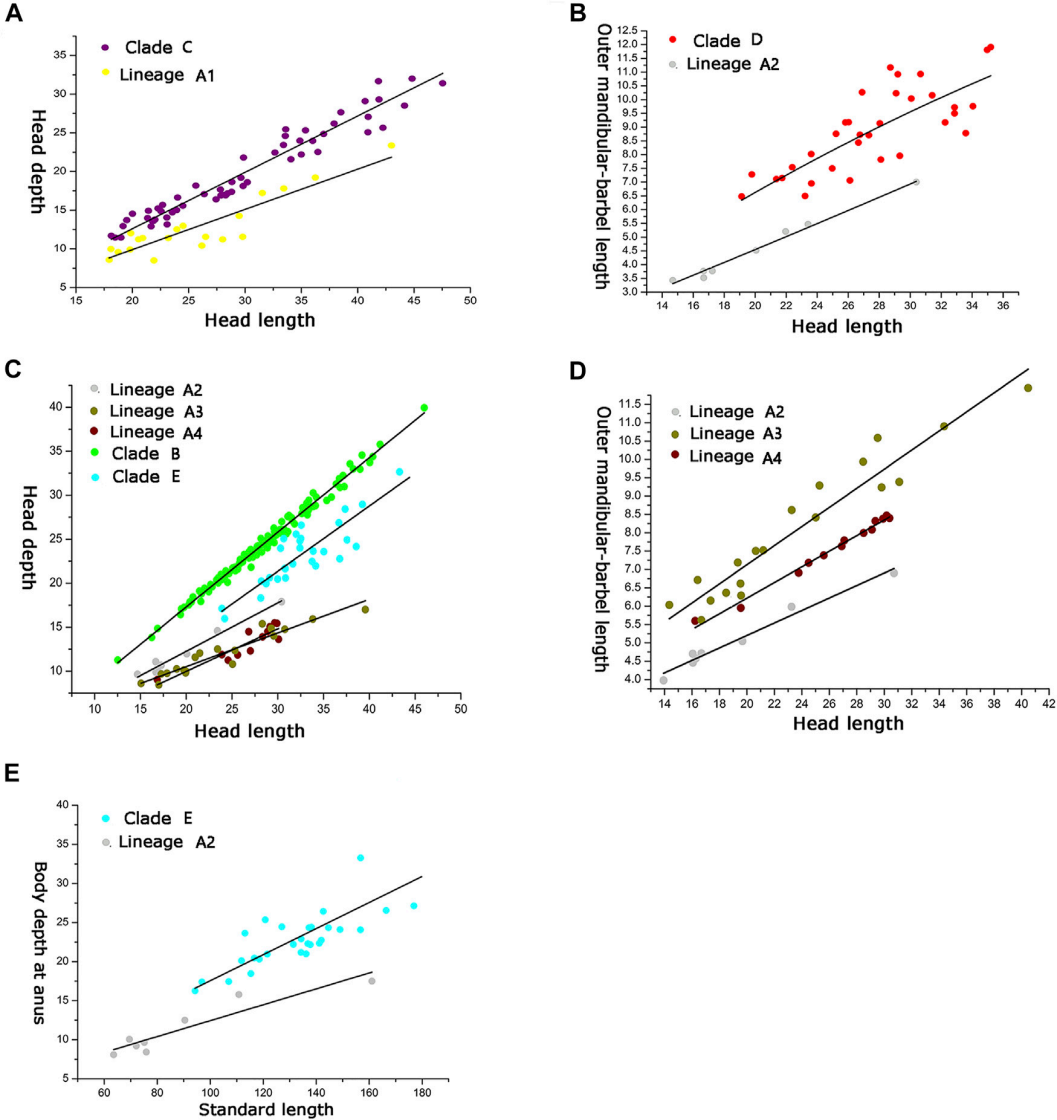
Relation between **(A)** head depth and HL for Clade C and Lineage A1; relation between **(B)** outer mandibular barbel length and HL for Clade D and Lineage A2; relation among **(C)** head depth and HL for Clades A and B, Lineages A2, A3, and A4; relation among **(D)** outer mandibular barbel length and HL for Lineages A2, A3, and A4; relation between **(E)** body depth at anus and SL for Clade E and Lineage A2.

Clade C and Lineage A1 differ from all other lineages in having more vertebrae numbers (47–49 vs. 39–46; Figure 5A), but both differ from each other in the head depth (58.4–76.1% HL vs. 43.6–55.6; Figure 6A). Clade D is separated from all other lineages except Lineages A2 and A3 in having fewer vertebrae (39–43 vs. 44–49; Figure 5A). It has a slight overlap in this character (39–43 vs. 43–45; Figure 5A) with Lineages A2 and A3 but differs from the former in having fewer anal-fin rays (15–18 vs. 19–21; Figure 5B) and from the latter in possessing longer outer mandibular barbels (length 24.4–36.8% HL vs. 21.1–23.9; Figure 6B). Clades B and E differ from Lineages A2-A4 in having a deeper head (depth 67.7–89.1% of HL vs. 43.16–66.4), and the head depth is greater in Clade B than in Clade E (Figure 6C). Lineage A2 differs from Lineages A3 and A4 in having shorter mandibular barbels (length 21.1–23.9% of HL vs. 23.53–34.0) and a deeper head (depth 58.6–66.4% of HL vs. 43.1–55.7) (Figures 6C,D), from Clade E in having a deeper body (depth at anus 11.1–14.2% of SL vs. 15.4–21.2) (Figure 6E). Mandibular barbels are longer in Lineage A3 than in Lineage A4 (Figure 6D).

## Discussion

### Hidden Diversity

The hidden species diversity of the morphology-based species *T. albomarginatus* s.l. is unveiled in this study. Nine distinct lineages are recovered in the phylogenetic analysis based on combined mtDNA (COI and Cyt. b) and nuclear (RAG2) genes, and the use of different molecular species delineation methods results in the detection of six or nine MOTUs (Figure 2). The mismatch in molecular species delineation utilizing these two methods (ABGD and PTP analysis) arises from the delimitation of Clade A as one or four MOTUs. It is not surprising that the ABGD method has a relatively conservative species delineation for the clade, given the small genetic divergences among constituent lineages (A1-A4). Previous investigations have demonstrated that the ABGD analysis, which is based only on nucleotide divergence to delineate species (Laris et al., 2015), fails to distinguish sister species that are easily recognizable from morphology (Lessios, 2008; Silva et al., 2018). In contrast, the PTP analysis requires neither an ultrametric input tree nor a sequence similarity threshold as input and thus outperformed the other methods when genetic divergences between species are small (Kapli et al., 2017). In the case of the *T. albomarginatus* s.l., Lineages A1-A4 are delineated by this method as four molecular operational taxonomic units (MOTUs), despite their small genetic divergences.

The nine lineages recovered here for *T. albomarginatus* s.l*.* correspond to eight geographic populations. Sympatric lineages D1 and D2, both indistinguishable morphologically, represent one geographical population. Each of the remaining seven lineages forms its own geographical one. These eight allopatric populations are distinguished by the combination of two meristic counts and three morphometric measurements (Figures 5, 6) and so represent eight operational taxonomic units (OTUs). Congruence in molecular and morphological evidence, coupled with concordant geographical distributions, supports the delineation of the nine lineages as eight putative species. It is evident that the currently recognized widespread species *T. albomarginatus* s.l. represents a species group including multiple species.

There is growing evidence for the existence of underestimated diversity when morphology-based species are scrutinized under a molecular phylogenetic context (Mongkolsamrit et al., 2020). Two or more distinct species are regarded to be cryptic if they are erroneously classified under one species name (Bickford et al., 2007). In most cases, cryptic species are those species that represent distinct lineages under molecular scrutiny but have been classified as a single species owing to failure to detect their subtle morphological differences (Jirsová et al., 2019). However, species of this kind should be referred to as pseudocryptic species (Matsuda and Gosliner, 2018) and cryptic species are truly represented by those species recently diverged, sympatrically occurring, and distinguishable only by DNA data (Brickford et al., 2007; Schnurr et al., 2018). Cases like this are also frequently seen in previous studies on freshwater fishes, but relatively common on invertebrates (Matsuda and Gosliner, 2018). Both cryptic and pseudocryptic species are herein unveiled within the *T. albomarginatus* species group. The species represented by the paired lineages (D1 and D2) are truly cryptic species, as both exhibit a distinct sequence divergence of 2.2% for the Cyt. *b* gene, a complete overlap in their ranges, and no external morphological differences. External morphology and allopatric distribution patterns distinguish the species represented by Lineages A2-A4 from the rest of this species group, and no previous studies have ever described them after a review of literature, thus making them pseudocryptic species.

### Speciation and Rapid Diversification

The *T. albomarginatus* species group forms a monophyletic assemblage under rapid diversification (Figure 2). The majority of genetic divergences are found within five clades (A-E), but with only a small fraction of the genetic variation distributed among them, which is generally considered as evidence for rapid diversification (Groman, 2010; Sasaki et al., 2010). Clade A also shows a lower resolution of phylogenetic relationships among the four constituent lineages; the phylogeny displays a reticulate cladogenesis in the haplotype network (Figure 3), thereby indicating a phenomenon of rapid radiation (Cornejo and Scheidegger, 2015). Despite the lower genetic divergences here detected among these four lineages, they are distinguishable from morphology (Figures 5, 6) and represent four putative species.

The divergence time estimation among lineages A1 to A4 ranges from 1.35 to 1.72 Ma (Figure 3B), which coincides with the uprising of the Wuyi Mountains that likely occurred during the mid-Pleistocene (Zhang et al., 1990; Chiang et al., 2013). The rapid diversification within Clade A can best be interpreted as ecological adaptations of its included species to running waters as a result of mountain uprising. Our morphological examination indicates that the number of vertebrae is the most variable trait in the meristic counts (Figure 5). More vertebrae counts are found in Lineage A1 than Lineages A2-A4 (Figure 4). Consistent with this is the elevated distribution of the former when compared with the latter. It is likely that the increase in the vertebrae count of the species represented by Lineage A1 is an adaption to running water (such as flow velocity) in the Min-Jiang basin. It has been revealed that the number of vertebrae is correlated with ecological adaptations in freshwater fishes (Kimura et al., 2012; Kiso et al., 2012). Morphometric measurements such as head depth, outer mandibular-barbel length, and body depth at anus, which exhibit marked differences in the *T. albomarginatus* species group (Figures 5, 6), have been found to be related to ecological adaptations (Slaughter and Jacobson, 2008; Zeng and Liu, 2011; Pledger et al., 2014).

Three clades (A, B, and C), inhabiting in the lower Chang-Jiang basin and coastal rivers of Southeast China, are phylogenetically allied to each other but exhibit low sequence divergence and low resolution of phylogenetic relationship, thus indicating an explosive speciation event. The emergence of the most recent common ancestor of these three clades was estimated to be about 3.91 Ma, and the divergence time between Clades B and C was roughly 3.45 Ma. This period is broadly consistent with the intense uplifting of the Qinghai–Tibetan Plateau and the accompanying occurrence of Asian monsoons during the mid-Pliocene (3.6–2.6 Ma; Zhisheng et al., 2001; Zheng et al., 2002). Tectonic movement and climate change, which reshaped the geographic configuration of current drainage basins, are hypothesized to be key driving forces of vicariant events leading to the allopatric distribution of these clades. The geographic separation of the Poyang Lake system from streams on the southern bank of the lower Chang-Jiang in Anhui and Jiangsu provinces, triggered by the uplift of the Huangshan and Tianmu Mountains, is likely responsible for the disjunct distribution of Clades B and C, and from coastal rivers basin of Fujian Province, brought about by the rising of Wuyi-Yuhuan Mountains for that of Clades A and C. The disjunct distribution between Clades A and B is attributed to the formation of the watershed between streams in Zhejiang Province and Fujian Province (Figure 1). The effects of river capture on the expansion and dispersal of fishes were greatly reduced with the declining East Asian monsoon and lowered precipitation during the mid-Pliocene (Sun and Wang, 2005). Similar scenarios of regional divergence in South China during this timescale have previously been studied in some fish species, e.g., *Hemiculter leucisculus* (Chen et al., 2017) and *Rhynchocypris oxycephalus* (Yu et al., 2014).

Allopatry is also observed in Clade E and the remaining clades as well as Clades D and A + B + C. Clade E, occupying the Liu-Jiang of the middle Zhu-Jiang basin, is the earliest lineage to diverge in the *T. albomarginatus* species group. The time that Clade E separated from the rest of this species group was estimated to be around 4.92 Ma. Evidently, the disjunct distribution between this clade and others results from the formation of a water division between the middle Zhu-Jiang and middle Chang-Jiang basins and particularly between Liu-Jiang and Xiang-Jiang; the vicariant event responsible for this allopatric pattern was triggered by the upsurge of the Miaoling Mountains during the Early Pliocene. The disjunct distribution of the paired clades D and A + B + C can also be plausibly explained by geographical isolation; namely, the formation of the watershed between the Dongting and the Poyang Lake resulted from the uprising of Luoxiao-Mufu Mountains. Sympatric Lineages D1 and D2 have probably experienced allopatric divergence and subsequent merging. Continuous interbreeding between the two populations would lead them to have no autapomorphy to be diagnosed, just as suggested by Mishler et al. (2000).

Clade B has the most widespread distribution across many river basins, with its most northern extent at the Dongping Lake in Shandong Province (belonging to the Huang-He basin). It exhibits a similarity of genetic structure (Figures 2, 3), therefore indicating ongoing gene flow and dispersal among these hydrographic basins. This was made possible *via* the Beijing-Hangzhou Grand Canal, which was constructed more than 2,500 years ago (Chen et al., 2010). The canal starts from the lower Qiantang-Jiang of Zhejiang Province, across the lower Chang-Jiang, lower Huai-He, and lower Huang-He, to the Hai-He. The creation of the Beijing-Hangzhou Grand Canal makes it possible to have a northward-extended distribution as it has presently, given the presence of its closely allied lineages in South China: A in coastal rivers of Fujian Province and C in the Poyang Lake system.

### Taxonomic Considerations

In this study, integrative taxonomy supports the detection of *T. albomarginatus* s.l. as including eight putative species. A conservative stance is adopted to recognize five taxa including four species, namely, *T. albomarginatus* (Clade B), *T. analis* (Clade C), *T. lani* (Clade E), and *T. zhangfei*, sp. nov. (Clade D), and a species complex (Clade A): the *T. similis* complex. Taxonomic confusions surrounding *T. albomarginatus* have been clarified by Cheng et al. (2021) based on their examination on the syntype and topotypes. The main characters typical for this species are shared with specimens from the lower reaches of the Chang-Jiang, Huai-He, and Qiantang-Jiang basins, and also coastal river basins of South Zhejiang Province. Samples from these rivers nested into Clade B in combined phylogenetic trees (Figure 2). Therefore, we have little doubt that Clade B represents the true *T. albomarginatus.*

The pairwise divergences of both the Cyt. *b* gene among Clades A-E range from 1.9 to 2.4%, which fulfill the barcode threshold of 2% (Ward, 2009) and are comparable to the pairwise divergence of 1.7% between *T. aurantiacus* and *T. ondon* (Ku et al., 2007). The substitution rate of the East Asian bagrid mitochondrial Cyt. *b* gene is estimated to vary from 0.18 to 0.30% sequence divergence per million years (Peng et al., 2002), much lower than that of other freshwater fishes, for example, 1.5% in salmonids (Shedlock et al., 1992), 0.68–1.0% for *Cobitis* species (Perdices and Doadrio 2001; Doadrio and Perdices 2005), 1.7% for *Candidia barbatus* (Wang et al., 2011), and 0.52–0.82% for cyprinid species (Zardoya and Doadrio, 1999; Ruber et al., 2007). This can explain, to a large extent, the relatively low sequence divergences here detected among five clades within the *T. albomarginatus* group when compared with non-bagrid species. The most recent common ancestor of *T. albomarginatus* group dates back to the early Pliocene when the complex paleogeographical history of East Asia, e.g., tectonic movement and climate change, facilitated the speciation of freshwater fishes. Thus, five main clades underwent recent rapid diversification, and so did the paired congeneric species *T. pratti* and *T. truncatus* (Figure 3B). Taking into account that Clade A was more recently diverged when compared with all other clades (in the middle Pleistocene), we follow the congruence of the time-calibrated frame to temporarily consider the A1-A4 lineages as a species complex.

Although the *T. albomarginatus* species group usually inhabits running waters of rivers, it has a limited swimming ability as indicated in their emarginated or round caudal fin instead of a deeply forked one in good swimmers such as *T. fulvidraco* which is widespread in mainstreams of East Asian rivers. The five taxa recognized here for this group are less widely distributed in tributaries of both the Chang-Jiang and Zhu-Jiang, but not found in their mainstreams; they also do not occur in the headwaters of the rivers they inhabit. Limited swimming ability and no occurrence in headwaters of nearby rivers indicate that inter-basin dispersal is highly unlikely, which leads to restricted gene flow between the clades. Diagnosable morphological differences detected in this study reinforce our confidence in the recognition of *T. albomarginatus* s.l. as containing multiple species.

**FIGURE 7:**
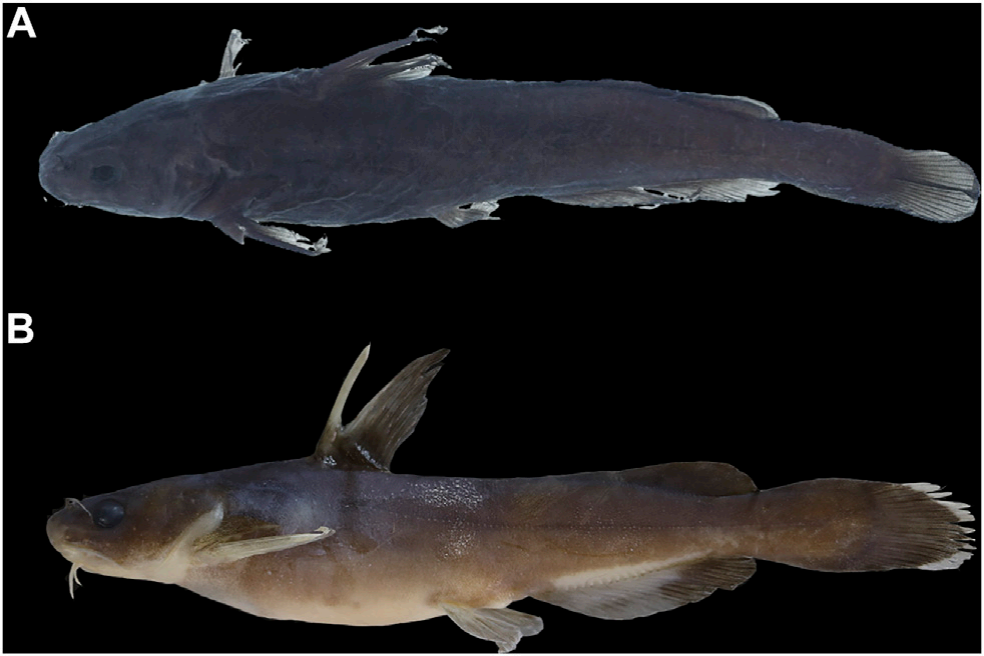
Lateral view of *T. analis* for: **(A)** AMNH 6421, hopotype; 101 mm SL; Hekou, Yanshan, Jiangxi Province, and **(B)** IHB201707014311, 124.2 mm SL, Yudu county, Jiangxi Province, in Gan-Jiang.

The available name for the species represented by Clade C is *Tachysurus analis*, originally described by Nichols (1930) based on a single specimen of 101-mm SL collected from Hokou, northeastern Kiangsi (now Hekou Town, Yanshan County, Jiangxi Province), in an affluent of Lake Poyang-the Xin Jiang. Despite the current identification of *T. analis* as a valid species (Chu et al., 1999), it is known merely by the type specimen. Seemingly, no additional specimens have been reported from the Xin Jiang or elsewhere until now. Our photograph examination on the type (AMNH 6421) confirmed that *T. analis*, as stated in its original description, has a narrow, slightly tapering, rounded caudal fin (Figure 7A), a character not shared with all other congeneric species. In the molecular phylogenetic trees (Figure 2), samples caught from the Xin-Jiang (type locality of *T. analis*), Fu-He, and Gan-Jiang of the Poyang Lake system clustered into Clade C which was distantly allied with Clade B, representing a species distinct from *T. albomarginatus*. These samples also have a narrow, slightly tapering, rounded caudal fin (Figure 7B), a character uniquely diagnostic for *T. analis*; therefore, we consider them conspecific with *T. analis*. The most likely explanation for no reports of *T. analis* collected from the Xin-Jiang or the Poyang Lake system since its original description is misidentification as *T. albomarginatus* due to the shared presence of a broad white mark along the distal caudal-fin margin.

Recently, Cheng et al. (2021) confirmed that specimens previously misidentified as *Tachysurus albomarginatus* from the Liu-Jiang of the Zhu-Jiang basin belong to a new species named as *T. lani.* Samples from this river were recovered in this study to unite into Clade E. It is evident that *T. lani* is the available name for the species represented by this clade. Clade D, including two paired lineages B1 and B2, deserves specific status, given the recognition of their close genetic cluster (Clade E) as a valid species and also marked differences in the counts of vertebrae and anal-fin rays (Figure 5) with all other clades. A new species, *T. zhangfei* sp. nov., is here described for Clade D (see below). In reality, the new species represents a cryptic species complex, within which two cryptic species having overlapping distribution and indistinguishable morphology are included (Lineages D1 and D2).

**FIGURE 8:**
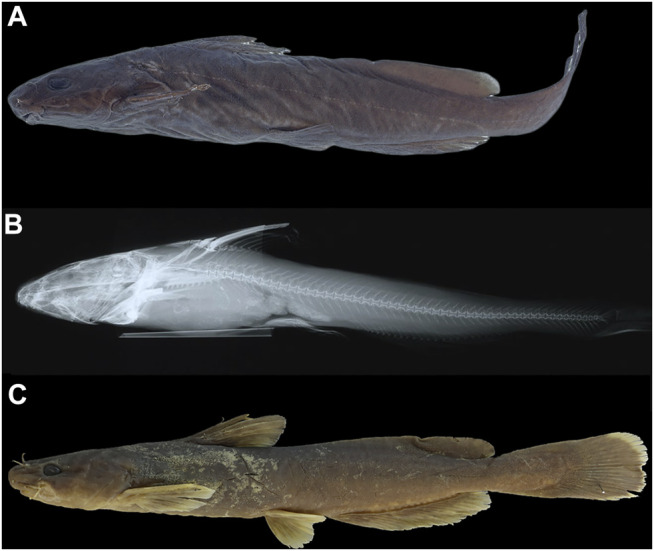
*Tachysurus similis*: AMNH 8444, holotype, 119 mm SL; Nanping, Fujian Province, in lateral view **(A)** and X-ray **(B)** of body. IHB201809021142, topotype, mm SL, 152 mm SL Nanping, Fujian province **(C)**.

*Tachysurus similis*, initially described by Nichols (1926) based on one specimen of 119 mm SL from the Min-Jiang, Yenping (now Nanping City in Fujian province) (Figure 8A), is the available name for the species represented by Lineage A1. This species has not been mentioned by subsequent researchers except Ferraris (2007), who listed it as species inquirenda in the Bagridae. The type (AMNH 8444) has 21 anal-fin rays rather than 17 as stated in the original description, and 47 vertebrae based on our observation on its X-ray photograph (Figure 8B). Our ongoing taxonomy studies of the currently recognized Chinese *Tachysurus* also show that *T. analis* is the only species with 47–49 vertebrae. Samples from Min-Jiang, with 19–22 anal-fin rays (Figure 5B) and 47-49 vertebrae (Figure 5A), nested into Lineage A1 and are distantly allied to Clades B and C (corresponding to *T. albomarginatus* and *T. analis*, respectively) in the phylogenetic trees (Figure 2). This clearly indicates that Lineage A1 represents a species distinct from either *T. analis* or *T. albomarginatus*. Therefore, the identity of specimens from the Min-Jiang previously misidentified as *T. albomarginatus* should actually be *T. similis*. Its validity has until now gone unrecognized, mainly owing to the imprecise original description, particularly in the count of anal-fin rays and the caudal-fin shape alike. The accompanied illustration (Page 1: Figure 1) depicts a species with a weakly forked instead of a slightly emarginate caudal fin (Figure 8C). To our knowledge, no specimens of *Tachysurus* have been found to possess 21 anal-fin rays and a weakly forked caudal fin. As such, the original description is likely erroneous.

As elaborated above, the three lineages (A2-A4), clustered within Clade A, represent three pseudocryptic species. However, it is premature to assign a specific status to each of these lineages, given that their sample sizes and number of sampling localities in this study are relatively small, especially for Lineages A2 and A3 (Figure 1). The names *T.* aff. *similis* Jin-Jiang, *T*. aff. *similis* Han-Jiang, and *T.* aff. *similis* Jiulong-Jiang are provisionally used to refer to the pseudocryptic species within the *T. similis* species complex*.*

### Biodiversity Conservation

The detection of unrecognized diversity of *Tachysurus albomarginatus* s.l. in this analysis has many implications for biodiversity conservation. The species, as conventionally defined, has so far been listed as LC (least concern) in the recent assessment of Chinese freshwater fish red list (Zhang & Cao, 2021), owing to its wide distribution. It, however, has been demonstrated here to be a species group that can be further split into four species and a species complex, each with comparatively narrower distributions. This restricted distribution, one of the main reasons for concern in maintaining unique species in biodiversity conservation (Barlow et al., 2010), justifies concern on the long-term viability of these species or species complex. *Tachysurus lani*, represented by Clade E from the Liu-Jiang, has merely been collected in two sampling locations (locality 33 and 34, geographically separated by more than 200 km; see Figure 1) during our field surveys. The population of the *T.* aff. *similis* Jin-Jiang, represented by Lineage A2, is found only in one sampling location, with fewer than one dozen specimens caught during our field surveys into this river from 2018 to 2020. *Tachysurus lani* and the *T.* aff. *similis* Jin-Jiang, just like other rheophilic and predatory fishes, can be put under severe threat from anthropogenic disturbances such as river damming and illegal fishing. Thus, more efforts should be dedicated to protecting them, given that the single or fragmented habitats and small population sizes can result in lowered evolutionary potential and elevated extinction risk in the wild due to low genetic diversity (Cooper and Walters, 2010).

The revelation of hidden diversity for *Tachysurus albomarginatus* s.l. has ramifications not only for the protection of the detected putative species but also for regional biodiversity conservation of South China. Now, conservation efforts have been misdirected by the current misperception of freshwater fish diversity in the lowlands of South China where it is considered to have a lower proportion of endemic fish species. However, in this study, the currently recognized *T. albomarginatus* s.l. has been shown to exhibit a high level of geographic divergence. This highlights the need not only for molecular scrutiny of widely distributed species in lowland South China, particularly river-flooding plains, but also for adjustments of biodiversity conservation strategies to protect this largely overlooked fish diversity. Globally, freshwater ecosystem protection is subject to a territorial ecosystem scheme that puts emphasis on forests and land vertebrates; only a small portion of freshwater ecosystems is under protection (Hazen and Harris, 2007; Crous, 2013). South China is not the exception; in the framework of priority areas of territorial biodiversity conservation of China (Ma et al., 2010), merely the Poyang and Dongting lakes and headwaters of some rivers are included, and a small portion of freshwater fishes are thus protected. It is in urgent need to reconsider the biodiversity conservation plan of South China, particularly for freshwater ecosystems that are totally overlooked or ignored.

It is worthy of pointing out that the *Tachysurus similis* complex has the higher level of regional endemism in Fujian Province where each of the four major coastal river basins has its own putative species. This regional endemism is more or less repeated in many fish species such as *Onychostoma barbatulum* (Jang-Liaw and Chen, 2013), *Acheilognathus macropterus* (Yang et al., 2011), *Sinibrama macrops* (Zhao et al., 2013), and *Squalidus argentatus* (Yang et al., 2013). This evidently indicates that the coastal river basins of Fujian Province are a potential biodiversity hotspot in China and the members of *T. similis* complex should be considered as separate conservation units. In conclusion, it is apparent that priority should be given to the freshwater fishes of the coastal river basins of South China in biodiversity conservation plans.

### Taxonomic Accounts

#### *Tachysurus zhangfei* sp. nov.

**FIGURE 9:**
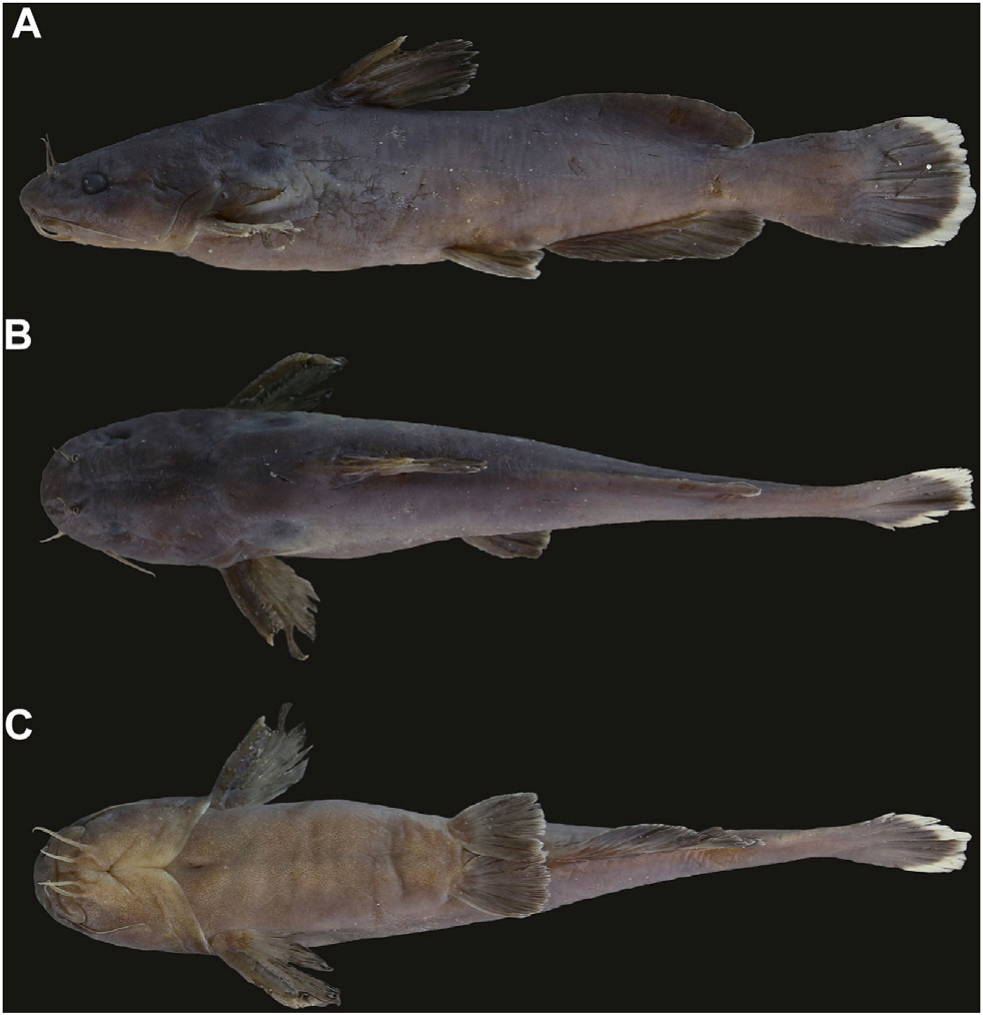
**(A)** Lateral **(B)** dorsal and **(C)** ventral view of *T. zhangfei* in freshly captured specimen of 128.0 mm SL, from Tongren city, Guizhou province.

(Figure 9; Supplementary Figure S3, and Table 1).

*Leiocassis albomarginatus* (not Rendahl, 1928): Ze, 1989: 249 (Jing-Jiang flowing into Yuan-Jiang of middle Chang-Jiang basin in Jiangkou County).

#### Holotype

IHB201709011724, 138.2 mm SL; South China: Guizhou Province: a stream tributary to Yuan-Jiang of middle Chang-Jiang basin in Songtao County.

#### Paratypes

IHB201709011717-23, 7 specimens, 76.6–154.2 mm SL; other data same as holotype.

#### Diagnosis

A member of the species group of *Tachysurus* defined by having a smooth anterior margin of the pectoral spine, short maxillary barbels not extending to the base of pectoral fin (Clade I of Ku et al., 2007), and an emarginate, truncate, or rounded caudal fin (Cheng et al., 2021: 12, Figure 10), with a broad white mark along the distal edge (Figures 7C, 8B, 9A). *Tachysurus zhangfei* is distinguished from all other species of this group by having fewer vertebrae (39–43 vs. 44–46 in *T. albomarginatus* and *T. lani*, or 47–49 in *T. analis* and *T. similis*; see Figure 5A), fewer anal-fin rays (15–18 vs. 19–21 in *T. lani* and the *T. similis* complex except for *T.* aff. *similis* Jin-Jiang or 20-23 in *T. analis*; see Figures 5A,B deeper body at anus (15.0–19.7 vs. 10.8–15.5% of SL in the *T. similis* complex; see Supplementary Figure S2A), a shallower head (depth 58.4–73.5 vs. 82.0–89.1% in *T. albomarginatus*; see Supplementary Figure S2B), and longer outer mandibular barbels (length 24.4–36.8 vs. 21.1–23.9% of HL in *T.* aff. *similis* Jin-Jiang, see Figure 6B).

### Description

Morphometric data in Supplementary Table S1. See Figure 9 and Supplementary Figure S3 for general body appearance. Dorsal profile rising gradually, but not steeply from snout tip to dorsal-fin origin, then sloping evenly from there to posterior end of adipose-fin base and increasing ventrally to dorsal origin of procurrent caudal-fin rays. Ventral surface of head flattened, ventral profile of body straight or slightly convex from pectoral-fin insertion to anal-fin origin, decreasing evenly from posterior end of anal-fin base to origin of ventral procurrent caudal fin rays. Lateral line complete, straight, mid-lateral in position. Vetebrae 5 + 38–43.

Head depressed, broad, and covered with thin skin. Snout blunt in dorsal view, longer than eye diameter. Eye large, elliptical, covered with thick membrane, antero-lateral in head, visible when viewed dorsally, but not ventrally. Mouth inferior, crescentic; upper jaw projecting forward past lower jaw. Teeth villiform, in irregular rows on all tooth-bearing surfaces. Premaxillary tooth plates broad, of equal width throughout. Dentary tooth plates arched, broadest at symphysis, narrowing laterally, of same width at symphysis as premaxillary tooth plate. Vomerine tooth plate unpaired, continuous across midline, slightly curved anteriorly, much narrower than premaxillary tooth plate. Gill opening wide, extending from posttemporal to beyond isthmus. Barbels in four pairs; nasal ones small, thread-like, extending slightly beyond midpoint of eye, and maxillary ones slender, extending beyond posterior margin of eye, almost to gill membrane. Mandibular barbels in two pairs, thick, short; inner barbels longer than eye diameter. positioned in transverse row at level of posterior naris, not extending beyond anterior margin of eye; outer barbels rooted posterolateral to inner mandibular barbell, extending beyond posterior margin of eye.

Dorsal fin with spinelet, spine, and seven soft, branched rays, inserted nearer to anal-fin origin than to snout tip or nearer to pectoral-fin insertion than to pelvic-fin insertion, closer to anal-fin origin than to snout tip. Spinelet flattened, with long, blunt distal tip. Dorsal-fin spine stout, with smooth anterior margin and slightly serrated posterior margin distally, longer than pectoral-fin spine. First dorsal-fin soft ray longest, surpassing tip of last ray. Distal margin of dorsal fin rays nearly straight. Nuchal plate triangular, with a pointed tip. Adipose fin inserted behind vertical through anal-fin origin, with convex distal margin for its entire length and a deeply incised posterior part to form a rounded apex. Adipose-fin base moderately long, longer than anal-fin base length.

Pectoral fin with a spine and eight soft branched rays, inserted anterior to vertical through posteriormost point of opercle, extending for half of distance to base of pelvic-fin spine. Pectoral-fin spine stout, sharply pointed at tip, shorter than dorsal-fin spine, with a smooth anterior margin and 12-15 (mean 13.3) strong serrations along posterior margin. Pectoral-fin margin straight anteriorly, convex posteriorly. Cleithral process triangular with a sharp pointed tip. Pelvic fin with five soft branched rays, inserted equidistant from vertical through caudal-fin base and tip of snout. Tip of depressed pelvic fin slightly extending beyond anal-fin origin. Pelvic-fin distal margin convex. Anus and urogenital opening halfway between anal-fin origin and pelvic-fin insertion.

Anal fin shorter than adipose-fin base in length, with 15 (4), 16 (12), 17 (9), or 18 (5) branched rays; inserted anterior to adipose-fin origin. Anal-fin origin nearer to caudal-fin base than to tip of snout. Distal margin of anal fin convex; anterior rays shortest.

Caudal fin broadly rounded or slightly emarginate, with 8 + 9 principal rays; upper lobe roughly equidistant from lower one; procurrent rays extending slightly anterior to fin base.

#### Coloration

In formalin-preserved specimens (Figure 9), body dark brown dorsally and laterally, grayish ventrally; caudal and anal fins blackish with a broad white distal margin, and other fins uniformly dark brown. When in life (Supplementary Figure S3A), body pale brown dorsally and laterally, yellowish ventrally; caudal and anal fins with a broad yellowish or white distal margin, and other fins uniformly brown.

#### Distribution

*Tachysurus zhangfei* is currently known from the Xiang-Jiang and Yuan-Jiang, and possibly Dongting Lake, in the middle Chang-Jiang basin.

#### Etymology

The specific epithet, used as a noun, is named after Zhang Fei, a character of the Romance of the Three Kingdoms—one of four famous literature works in China, in allusion to the presence of a blackish or brown body. He is endowed with an imagination of a black face in China opera.

#### Taxonomic Notes

The taxonomic usefulness of the presence or absence of serrations along the anterior margin of the pectoral spine and length of maxillary barbels was suggested in Ku et al., 2007 phylogenetic analysis of *Pseudobagrus* (=*Tachysurus*) predicated on mitochondrial sequence data. Clade I (Page, 156: Figure 1) recovered by them was formed by species with a smooth anterior margin of the pectoral spine and short maxillary barbels not extending to the base of the pectoral spine. Twenty-one constituent species can be subdivided into two groups based on the caudal-fin shape (Cheng et al., 2021). The species group with an emarginate, truncate, or rounded caudal fin, to which eight putative species recovered here for *Tachysurus albomarginatus* are referred, includes the following nine species: *T. adiposalis*, *T. brachyrhabdion*, *T. gracilis*, *T. omeihensis*, *T. tenuis*, *T. trilineatus*, *T. truncatus*, and *T. ussuriensis*. All these species except *T. adiposalis* and *T. omeihensis* were included in the ingroup of our molecular phylogenetic analysis. The topology of the phylogenetic tree (Figure 2) shows the monophyletic nature of the *T. albomarginatus* group. It is apparent that the caudal-fin coloration can be used as a character uniquely diagnostic for this species group. A narrow white mark along the distal margin of the caudal fin is present in *T. tenius* and *T. ussuriensis* (Cheng et al., 2021). A short narrow white mark is seen along the upper/lower margin of the caudal fin in both *T. brachyrhabdion* and *T. gracilis* (Li et al., 2005; Cheng et al., 2008).

## Data Availability

The datasets presented in this study can be found in online repositories. The names of the repository/repositories and accession number(s) can be found in the article/Supplementary Material.

## Supplementary Material

Supplementary Figure 1 Principal components analysis (PCA) scores of two sympatric lineages belonging to Clade D.

Supplementary Figure 2 Relation between **(A)** body depth at anus and SL for *T. zhangfei* and the *T. similis* complex; relation between **(B)** Head depth and HL for *T. zhangfei* and *T. albomarginatus*.

Supplementary Figure 3 Lateral **(A)**, dorsal **(B)** and **(C)** ventral view of *Tachysurus nicholsi* sp. nov., IHB 201709011724, holotype, 138.2 mm SL.
