# Supplementary figures and images for: Eight in One: Hidden Diversity of the Bagrid Catfish Tachysurus albomarginatus s.l. (Rendhal, 1928) Widespread in Lowlands of South China
Source: Front Genet. 2021 Nov 17;12:713793. doi: 10.3389/fgene.2021.713793 (PMC8635968; doi:10.3389/fgene.2021.713793)

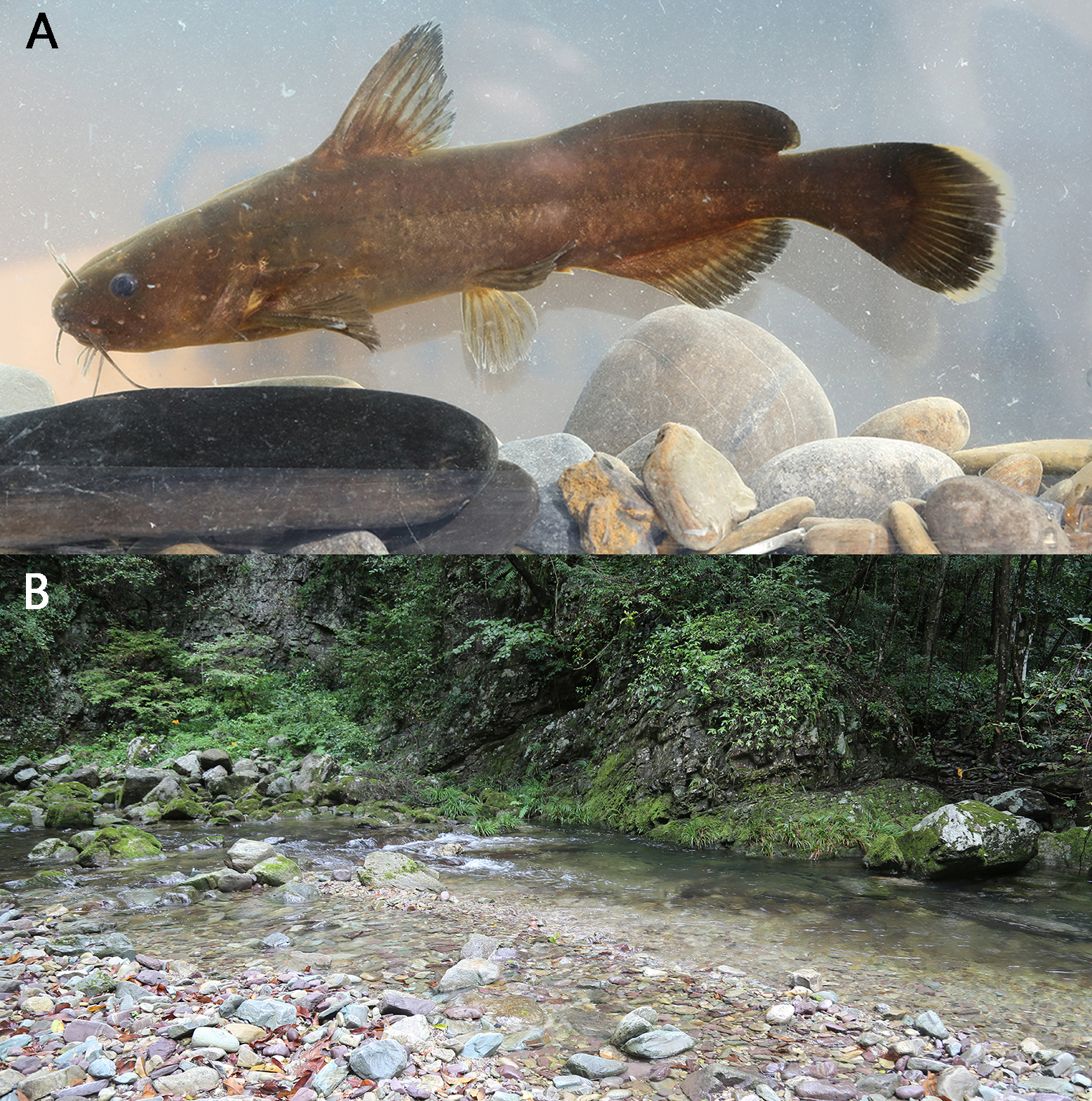

Supplement: Supplementary file 2 [file Image3.JPEG]

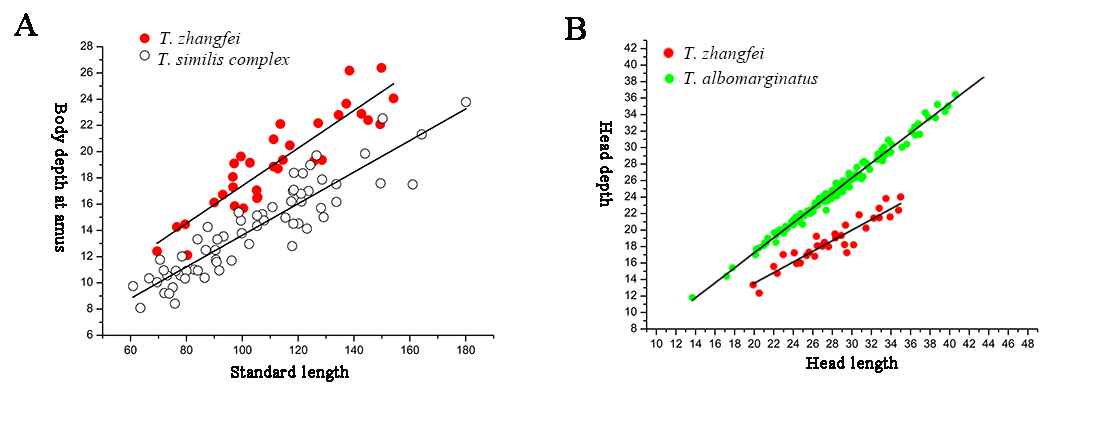

Supplement: Supplementary file 4 [file Image2.TIF]

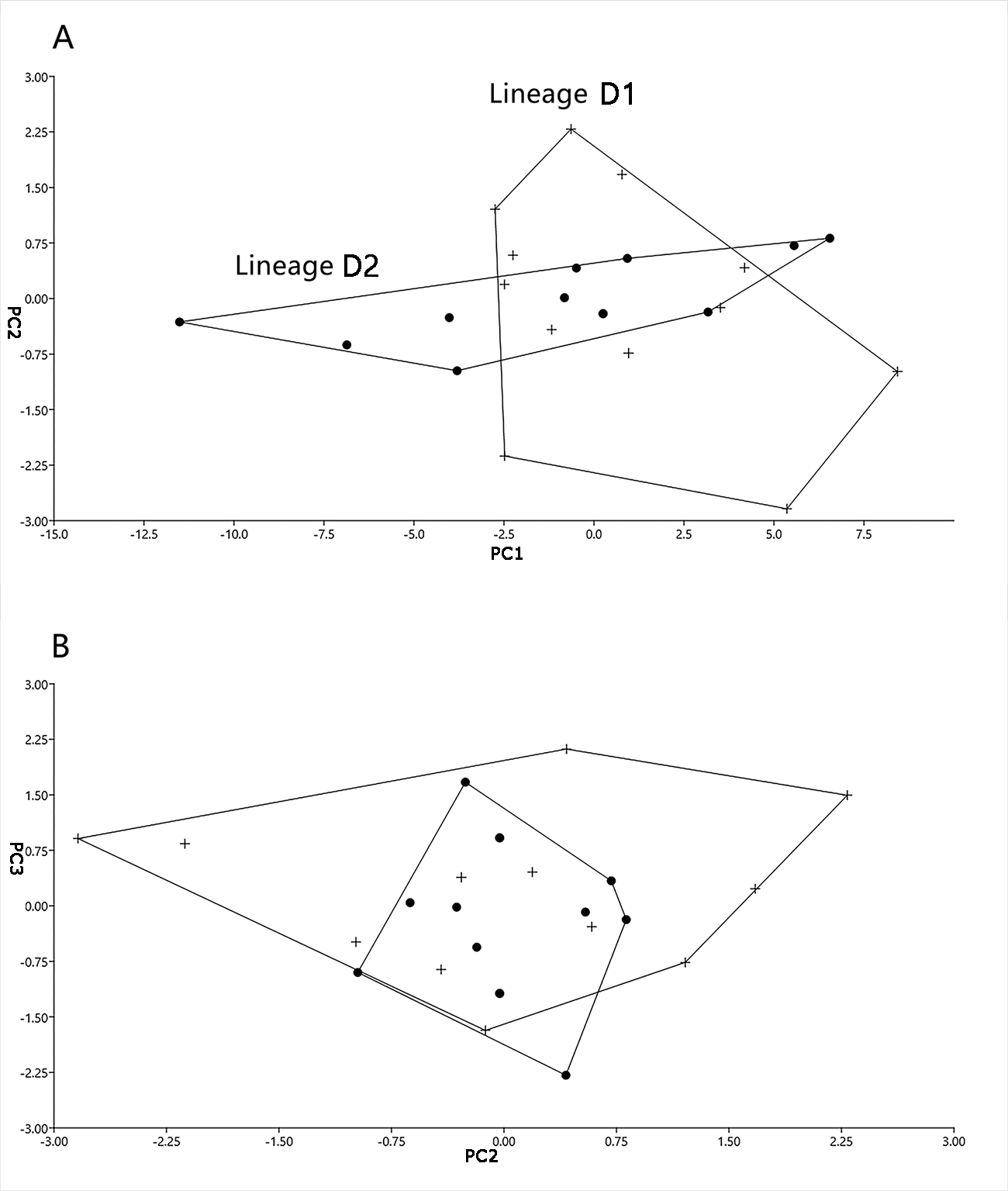

Supplement: Supplementary file 5 [file Image1.TIF]
