# Supplementary material for: Eight in One: Hidden Diversity of the Bagrid Catfish Tachysurus albomarginatus s.l. (Rendhal, 1928) Widespread in Lowlands of South China
Source: Front Genet. 2021 Nov 17;12:713793. doi: 10.3389/fgene.2021.713793 (PMC8635968; doi:10.3389/fgene.2021.713793)
Supplement: Supplementary file 3 [file Table1.DOCX]

**Supplementary table 1.** Morphometric data for *Tachysurus zhangfei* sp.nov..

|  | *Tachysurus zhangfei* sp.nov. | | |
| --- | --- | --- | --- |
|  | Holotype | Paratypes (n=7) | |
|  |  | Range | Mean±SD |
| Standard length | 138.4 | 76.6-154.2 | 112.5±5.1 |
| %SL |  |  |  |
| Body depth at anus | 18.9 | 15.0-19.7 | 16.8±1.2 |
| Predorsal length | 35.2 | 33.6-40.0 | 36.4±1.7 |
| Preanal length | 62.5 | 61.6-70.8 | 64.5±3.2 |
| Prepelvic length | 50.8 | 49.5-57.0 | 53.7±2.2 |
| Prepectoral length | 21.2 | 20.1-25.0 | 22.1±1.7 |
| Length of dorsal-fin spine | 16.7 | 15.6-22.8 | 19.1±2.4 |
| Length of dorsal-fin base | 10.7 | 9.9-13.2 | 11.0±1.6 |
| Length of pectoral-fin spine | 14.7 | 13.0-21.4 | 17.6±2.1 |
| Pelvic-fin length | 12.3 | 11.1-15.0 | 13.2±1.2 |
| Length of anal-fin base | 20.5 | 17.2-24.3 | 20.8±2.4 |
| Height of adipose-fin | 4.7 | 3.9-6.5 | 5.1±0.6 |
| Adipose to caudal distance | 15.3 | 10.1-14.9 | 13.9±1.3 |
| Length of caudal peduncle | 17.1 | 11.5-16.7 | 14.8±1.4 |
| Depth of caudal peduncle | 8.1 | 7.0-9.0 | 7.7±0.5 |
| Head length at latera | 23.3 | 22.4-27.5 | 24.7±1.3 |
| %HL |  |  |  |
| Head depth | 64.6 | 56.9-73.5 | 67.5±4.2 |
| Head width | 79.9 | 72.4-85.6 | 81.3±2.2 |
| Snout length | 33.9 | 29.7-36.8 | 32.8±2.8 |
| Interorbital width | 39.4 | 31.2-40.5 | 37.8±1.8 |
| Eye diameter | 21.7 | 12.6-19.2 | 18.8±1.5 |
| Mouth width | 43.4 | 35.6-48.5 | 40.2±2.9 |
| Length of nasal barbel | 20.6 | 21.0-34.4 | 24.5±3.1 |
| Length of maxillary barbel | 42.7 | 39.5-61.2 | 50.4±4.9 |
| Length of inner mandibular barbel | 18.9 | 15.3-26.8 | 21.4±2.3 |
| Length of outer mandibular barbel | 28.4 | 24.4-37.6 | 30.6±4.2 |
